# Annotations

**Published:** 1896-06-20

**Authors:** 


### Annotations.

### The Morphia Habit.

The death of Professor Middleton has drawn renewed attention to the dangers connected with the habitual use of opium, and especially to the risk of poisoning by the accidental administration of an excessive dose, which more particularly attaches to the self-administration of the stronger forms of the drug. The evidence obtained by the Opium Commission confirmed what had been stated before by many observers, that the regular use of a small quantity of opium is by no means that deleterious thing that some have imagined it to be, and in fact that it is taken by a large number of people in India exactly as the Englishman takes his daily glass of beer; the chief difference being that an occasional excess in opium is far less injurious, and certainly far less vexatious to the neighbourhood, than an occasional excess in alcohol. It has been said, indeed, by some, not altogether without wisdom, that alcohol and opium are the oil by which the machinery of life is lubricated, and friction between man and man made bearable. Both alike, however, must be used with judgment—temperately and without excess. The danger of opium lies in the facility with which it can be taken without drawing attention to the process; in the readiness with which it can be carried about so as to be available at any moment when temptation may arise; and on the lack of self-control induced by its use which leads to rapid increase of the dose to dangerous proportions, and, of course, the stronger the preparation the greater the danger. We would lay it down broadly that if opium is to be taken it should be taken openly, and that the dose should always be administered by someone else, not by the patient. Friends and relatives will often, no doubt, hesitate to give a dose which they know should not be taken, although they might not hesitate at all to repeat a dose of whiskey. They need not, however, load themselves with reproaches, or consider that they are making themselves accessory to what is wrong, for unless a man is absolutely confined to bed nothing that bystanders can do will prevent a morphinomaniac getting at his morphia; and by measuring out his quantum instead of allowing him, with his trembling hand and uncertain will, to dose himself, it at least is possible to prevent the most common cause of accident in such cases, viz., the unpremeditated taking of an over-dose.

### Sewage Farms on Trial.

There is no royal road to healthy sewage disposal, any more than there is to learning. Large and small towns have no other way of finding out what is healthiest and best but the way of experience and science. We put experience first, for in these cases experience is the guide and helper of science. The village of Wycombe Marsh and the contiguous borough of Chipping Wycombe have just furnished some object-lessons of a very practical kind on the sewage farm question. The farm in use by the borough of Chipping Wycombe borders on the village of Wycombe Marsh, and there is a river running near, into which some of the effluent from the farm is discharged. Last year the river banks were damaged, and much of the water overflowed on to the lower ground of Wycombe Marsh, and apparently found its way into the wells of the village. The result has been tremendous. In nineteen of the houses of the devoted village no fewer than twenty-four cases of typhoid fever showed themselves. Of deaths there were only two in the twenty-four cases; and this strongly confirms the supposition that it was the overflow from the river which polluted the wells; because, of course, the sewage effluent would be much diluted by the running water of the river, and that again by the water in the wells, and so only a highly diluted poison could be in operation. The poison was sufficient to produce typhoid, though not of a very fatal kind. Two conclusions emerge, and they are these: first, that sewage farming demands a high degree of skill and care in order that non-poisonous effluents only may be turned into running streams; and secondly, that village wells which are in any close contiguity to sewage farms should, if possible, be closed, and an adequate supply of water obtained elsewhere. Civilisation must, of course, get rid of its sewage; and sewage farms, when scientifically and conscientiously managed, are excellent methods of sewage disposal. But it is probable that in no long time they will have to be submitted to competent periodical inspection by a central authority in order that such calamities as that which has overtaken Wycombe Marsh may, as they ought to, become impossible.

### Summer Suicides.

Dreary November has hitherto enjoyed the "bad eminence" of being considered the most suicidal month in the year; but, according to a Blue-book just issued from the Home Office, it is July, and not November or December, which may claim that unenviable distinction. "Attempted suicides," we are informed, "occur in July with far greater frequency than at the beginning or end of the year." It is worthy of note that the very same thing happens in France. It is probable, also, that all civilised countries would show agreement with England and France in this particular. Naturally, one is led to think of the reason or reasons. The conclusion is that they must be physical and physiological rather than mental, and due to unfortunate circumstances. It is a well-known fact that in July, in consequence of the great heat and the stagnant condition of the atmosphere, the physical vitality is generally very low and the depression of the nervous system has reached its deepest. No laboratory experiments are required to prove these contentions. Everybody is aware that in July he is for the most part "played out," and is looking forward to his annual holiday with eagerness. An important question is whether or not anything can be done to combat the natural depression of July. No doubt the earlier taking of the summer holiday would be a step in the right direction; only if a month be taken at midsummer a further fortnight will be needed early in October, and that is costly in a double sense. The most practicable remedy for July depression is a suitable nerve tonic; and perhaps the best and most easily obtainable of all nerve tonics for summer weather is the sluicing of the head and spine with cold water every day, or even twice a day. If any man who is disposed to commit suicide will give himself a preliminary shower bath, it is highly probable that he will change his mind before the "towelling" is finished.

